# The Anti-Inflammatory Action of Pulsed Radiofrequency—A Hypothesis and Potential Applications

**DOI:** 10.3390/medsci11030058

**Published:** 2023-09-10

**Authors:** Menno E. Sluijter, Alexandre Teixeira, Kris Vissers, Luis Josino Brasil, Bert van Duijn

**Affiliations:** 1 Pain Medicine Center, Swiss Paraplegic Center, Guido A. Zäch-Strasse 1, 6207 Nottwil, Switzerland; 2 Clinica de Dor, R. São João de Brito 610, 4100-455 Porto, Portugal; alteix@gmail.com; 3 Department of Anesthesiology and Palliative Care, Radboud Medical Center, Geert Grooteplein Zuid 10, 6525 GA Nijmegen, The Netherlands; kris.vissers@radboudumc.nl; 4 Department of Anesthesiology and Pain Medicine, University of Porto Alegre, Porto Alegre 90050-170, Brazil; ljbrasil@yahoo.es; 5 PBDL, Institute Biology, Leiden University and Fytagoras BV, Sylviusweg 72, 2333 BE Leiden, The Netherlands; 6 Science Department, University College Roosevelt, P.O. Box 94, 4330 AB Middelburg, The Netherlands

**Keywords:** pulsed radiofrequency, oxidative stress, RedoxPRF, redox equilibrium, inflammatory diseases, age-related macular degeneration, major depressive disorder, stroke, chronic kidney disease, COPD

## Abstract

In 2013, it was reported that pulsed radiofrequency (PRF) could be applied to obtain a systemic anti-inflammatory effect. Patients with chronic pain and patients with an inflammatory condition from other disciplines could potentially profit from this finding. At that time, intravenous application was used, but since then, it became clear that it could be applied transcutaneously as well. This procedure was named RedoxPRF. This can be used both for regional and for systemic application. Recently, the basic element of the mode of action has been clarified from the analysis of the effects of PRF on a standard model of muscle injury in rats. The objective of this paper is to present a hypothesis on the mode of action of RedoxPRF now that the basic mechanism has become known. Cell stress causes an increased production of free radicals, disturbing the redox equilibrium, causing oxidative stress (OS) either directly or secondarily by other types of stress. Eventually, OS causes inflammation and an increased sympathetic (nervous) system activity. In the acute form, this leads to immune paralysis; in the chronic form, to immune tolerance and chronic inflammation. It is hypothesized that RedoxPRF causes a reduction of free radicals by a recombination of radical pairs. For systemic application, the target cells are the intravascular immune cells that pass through an activated area as on an assembly line. Hypothesis conclusions: 1. RedoxPRF treatment works selectively on OS. It has the unique position of having a point of engagement at the most upstream level of the train of events. 2. RedoxPRF has the potential of being a useful tool in the treatment of inflammatory diseases and possibly of stage 4 cancer. 3. In the treatment of chronic pain, RedoxPRF is an entirely new method because it is different from ablation as well as from stimulation. We propose the term “functional restoration”. 4. Controlled studies must be conducted to develop this promising new field in medicine further.

## 1. Introduction

**Figure 1:**
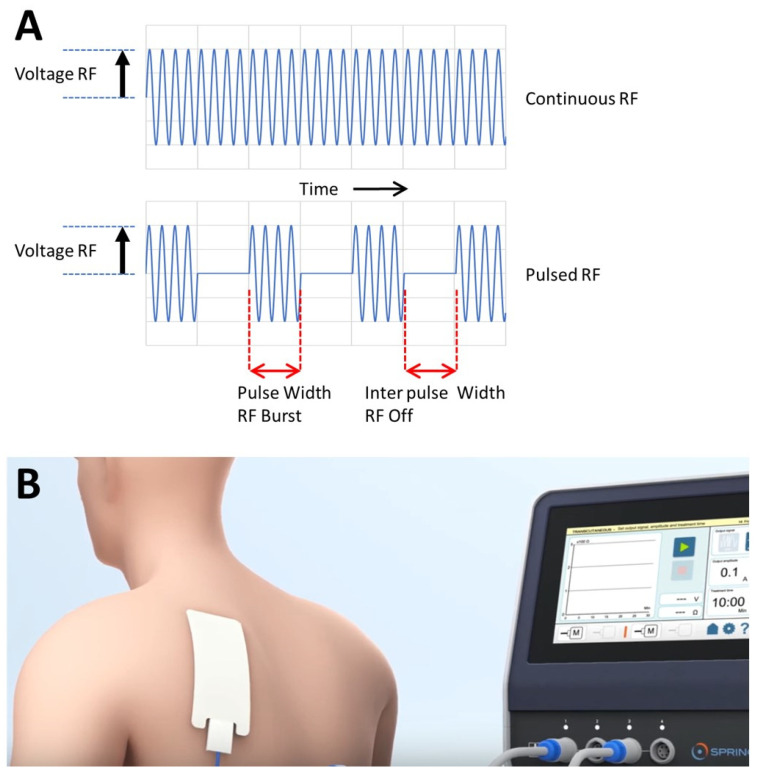
(**A**). The waveforms of continuous RF (CRF) and pulsed RF (PRF). While CRF is applied continuously without any resting phase, PRF has a (long) resting phase between the (short) electrical stimulations. RF—radiofrequency. The pulsed voltage amplitude determines the amplitude of pulsed RF current. Parameters which can be varied in the application of PRF are generally the voltage amplitude, the frequency within the RF burst, the RF burst width and the interpulse width. (**B**). Illustration of the application of skin electrodes attached to a modern digitized PRF generator for transcutaneous PRF treatment.

The invention of pulsed radiofrequency (PRF) [1] provides a nonthermal technique for minimally invasive treatment of pain as a potential substitute for the already existing ablative heat lesions with continuous radio frequency (RF). PRF currently is mainly used for the treatment of chronic pain in five indications: radicular pain, trigeminal neuralgia (TN), occipital neuralgia, shoulder, and knee pain [2]. The PRF mode of action was not known at that time, but this has not prevented its development into a widely used and frequently studied method. PRF involves application of pulsed electric fields, and it is a variation of continuous RF treatment (Figure 1A), which is believed to function by the application of heat [3]. PRF treatment, however, does not allow temperatures to run above 42 °C. The PRF pulsations avoid the risk of thermal tissue injury and, instead, expose the surrounding tissues to RF electric fields [4,5].

About ten years after the PRF invention, it was observed that intra-articular PRF could have satisfactory results even if the needle had been positioned at considerable distance from the afferent nerves [6]. From these results, the idea, preceding the hypothesis described in this paper, was formed that a PRF effect on immune cells causing an anti-inflammatory effect could be possible. Further confirmation of this effect (PRF effects distant from the application site) would open the way for the use of PRF, with a suitable application resulting in a systemic effect, for the treatment of the inflammatory diseases besides the more traditional use in pain treatment. Based on this insight, first trials with intravenous application were performed and published in four case reports [7]. These reports gave the method more attention, resulting in a multidisciplinary study on systemic PRF. This greatly contributed to the technique of applying systemic PRF by performing a finite elements computer study on the spread of the current showing that electromagnetic currents easily pass through a blood vessel wall [8] (performed at Radboud University Medical Centre in Nijmegen, the Netherlands). Consequently, the intravenous application could be replaced by a transcutaneous technique (Figure 1B). Besides this modeling work, in recent years, more became clear about the PRF’s working mechanism from experimental work. PRF treatment of monocytes in tissue culture increased TNFα secretion [9], and PRF treatment of astrocytes shows strong increases in the expression of genes involved in immune system modulation and, specifically, genes with anti-inflammatory functions [10]. Importantly, PRF shows that it can revert oxidative stress related to immune functions, and likely, the magnetic field component of PRF plays an important role in this [11].

## 2. The Hypothesis

**Figure 2:**
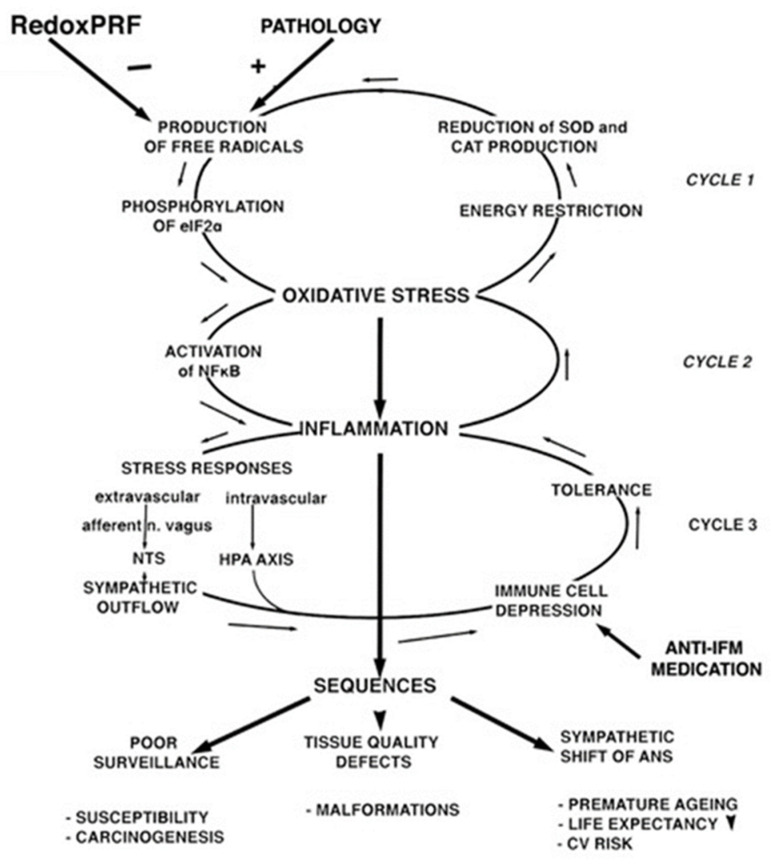
The three cycle mechanisms that are initiated by cell stress. See text for explanation.

In view of the recent above described experimental results, observations on patients treated, and theoretical considerations, all shaping the insights into the mode of action of PRF, we undertook to formulate a detailed hypothesis. The below described hypothesis involves different phases related to the cell-stress-initiated cycles of oxidative stress and inflammation (as shown in Figure 2). In this paragraph, we discuss the different parts of the hypothesis related to these cell-stress-initiated cycles.

### 2.1. The Reversal of the Free Radicals (FRs) Negative Cycle

We postulate that during PRF treatment the electric fields do not have any biological effect but that the very small magnetic fields cause a recombination of radical pairs [11], thus reversing the direction of cycle 1 of the cell-stress-initiated oxidative stress and inflammation cycles (as shown in Figure 2). Since this cycle is self-reinforcing, this reversal can also be expected to be effective at an early stage by increasing the production of superoxide dismutase (SOD). This could explain the fast response to systemic PRF where intravascular immune cells apparently react within their short trajectory through the activated area.

### 2.2. The Inflammatory Phase

The inflammatory phase is a natural response of the immune cells that have regained their normal reactivity to the overdue maintenance that was caused by the energy saving rules during the OS period. This phase confirms the findings of a laboratory study on the subject [9].

### 2.3. The Anti-Inflammatory Phase

It has become more and more apparent that epigenetic modifications in many different types of (immune system-related) cells play an important role in inflammation modulation (e.g., [12,13]). Therefore, we postulate that the PRF-induced improved condition of the cell is coded in the epigenome. This would explain the long periods of improvement that we have observed. Apparently, the epigenome only corrects its coding if there is a clear necessity to do so. The periods, therefore, vary in length according to the pathology. It is short in cancer patients—probably 2–4 weeks—and long in antitrypsin deficiency patients: 4–6 months.

## 3. Cell Stress and Its Consequences Related to the Hypothesis

There are several types of cell stress, for example, oxidative stress (OS), toxic stress like in diabetes type 2 (DT2) [14], and Endoplasmic Reticulum (ER) stress [15] like in diseases that are caused by a gene defect. Different types of stress are known to promote each other [16]. For example, toxic stress and ER stress may promote OS. In reverse, an effective treatment of OS may partly relieve symptoms that were caused by a different stress type.

OS starts with an overproduction of free oxygen radicals and some other radicals (FRs), causing a shift of the redox equilibrium to the oxidative side. This equilibrium is closely monitored in the cell [17]. Deviations cause phosphorylation eukaryotic induction factor eIF2α by one of the four members of the eIF2α kinase family, the hemeregulated inhibitor kinase (HRI) which is usually triggered by increased levels of ROS and transfer of the control over from the transcription program to the epigenome [18]. The cell is then, per definition, in OS. This in turn initiates three consecutive negative cycle mechanisms that are summarized in Figure 2. Cycle 1 is completed with the program of energy saving that is forced upon the cell by OS. This causes a reduction in the production of SOD and catalases, turning a proportional response into a self-reinforcing, positive feedback response.

OS can exist independently for longer periods of time [19]. It is only when the period of stress gets too long or too overwhelming that nuclear factor kappa B (NFκB) is activated, initiating inflammation (IFM). Cycle 2 is formed by OS and IFM promoting each other (Figure 2).

What happens next depends on dynamic interactions. If there has been an acute insult like a stroke or multitrauma, the response is an immune paralysis that is mediated by a massive sympathetic system activity and by the hypothalamic–pituitary–adrenal axis (HPA axis) response [20,21]. If the insult is lower grade, the reduction in the immune reactivity is partial. This leads to cycle 3, characterized by immune tolerance and by chronic inflammation at a level that is dictated by the level of tolerance (Figure 2).

These cycles underly the basic pathology of inflammatory diseases. The chronic IFM may be symptomatic or asymptomatic. For example, chronic glomerulonephritis has been named the “silent disease” because when the disease becomes symptomatic, it is often in its final stage. In contrast, in diseases like osteochondritis, COPD, and eczema, IFM is the prominent symptom from the early stages onwards. In these conditions, IFM may be so prominent that it is regarded as the primary pathology. We suggest that this is a wrong conclusion because the true culprit is not the IFM but the overproduction of free radicals which is several steps upstream. In our view, IFM is, therefore, a bottom-line result as opposed to an independent entity.

This is consequential if IFM is treated with anti-inflammatory medication. This will depress the immune reactivity even further and this may cause a further deterioration of the consequences that are shown in Figure 2.

## 4. Some Notes on Pulsed Radiofrequency (PRF) Related to the Hypothesis

### 4.1. PRF Application in Inflammation Is Called RedoxPRF

To avoid confusion with the “classical PRF” as it is used in invasive pain treatment, it is preferred to use another name for the alternative use of the method (e.g., intravenous or transcutaneous). We proposed to use the name “RedoxPRF” to accentuate the views on the mode of action, as reflected in the hypothesis.

Classically, the pulse in PRF is regular (fixed frequency), however, there is an increasing number of publications in the literature indicating that irregular rhythms accentuate biological effects [22,23]. For this reason, an irregular pulse version of the RedoxPRF current may be interesting. It is presently unknown if this irregular pulse frequency, which we named after the first users “Slujter–Teixeira-Puls (STP) current”, is more effective in this particular case. A device providing this option is available and could be used to investigate the advantage of the irregular pulse frequency.

Both the regular and irregular PRF have a short duty load of 15 msec/s. Therefore, in application, the equilibrated temperature stays below 41 °C for voltages not exceeding 45 V even during very low perfusion values [4,5]. RedoxPRF currents may, therefore, also be used for invasive PRF without temperature measurement.

### 4.2. Types of Treatment

It is the aim of RedoxPRF to expose cells in a tissue compartment to electric fields in the physiological range of 50–250 V/m. From our experience, this can be achieved with two approaches. In regional procedures, both resident immune cells and all other cells in the tissue compartment are exposed.

In systemic procedures, the goal is to obtain a systemic effect, such as a shift in the autonomic nervous system, by performing a limited strategic procedure. An example is the systemic procedure with plates on the lower and upper arm over the axillary artery that we explored as an alternative to intravenous PRF. In that case, intravascular immune cells are the apparent target when they move through an activated tissue compartment as on an assembly line. Not all immune cells pass through the area in the 15 min treatment time, but it nevertheless seems to be an effective procedure.

In another way to initiate a systemic effect, the trunk is taken as a huge tissue compartment and plates are placed just above the symphysis and on the back of the neck. In this treatment, a systemic effect was observed too, even when using modest currents that generate electric fields of not more than 100 V/m (see below, Section 4.3). This application has the advantage that the effect is likely not limited to the passing of immune cells.

### 4.3. Physical Aspects of RedoxPRF

During transcutaneous application, the system impedance is produced by the two skin plates and by the tissue impedance. The skin plate impedance varies considerably between manufacturers. A pair of medium-sized skin plates may contribute up to 60 Ohm to the total impedance, whereas the tissue impedance may be of the same order of magnitude, especially in procedures that involve the trunk. The voltage may, therefore, vary appreciably for a given current. Since the current determines the elicited electromagnetic fields, it follows that in transcutaneous application, the required current has to be determined before treatment is started. The electric fields are uniformly low in RedoxPRF, in contrast with invasive PRF that generates fields up to 250.000 V/m in a very thin layer around the needle tip. For the use of PRF equipment for RedoxPRF applications and the exact voltage–current settings in combination with the position and type of (plate) electrodes used, the manuals of the different equipment should be consulted.

### 4.4. General Information on RedoxPRF Treatment from Practice Observations and Considerations

RedoxPRF treatment does not cause any sensations. An exposure time of 15 min has been mostly used [7]. The method has one caveat and one contraindication. A caveat for diabetes type 2 (DT2) is justified because DT2 is an inflammatory disease Therefore, RedoxPRF treatment may cause an effect in patients with DT2 that has caused secondary OS. The effects can, therefore, vary from nil in mild DT2 to very large in decompensated DT2. Diabetic patients should be warned accordingly.

The contraindication concerns patients who have suppression of the immune system as part of their medical treatment, such as patients with an organ transplant.

In patients with OS, RedoxPRF is, according to the hypothesis, inducing an inflammatory phase that could be followed by heart rate variability measurements [24]. If the patient is in a normal condition, we have seen that this phase is short, ending before treatment is completed. It is reasonable to assume that this phase requires a significant amount of energy. In patients with advanced cancer, this may be a problem, and this can be anticipated by limiting the size of the tissue compartment to be treated and/or by shortening the treatment duration.

As a consequence of the RedoxPRF treatment, it could be expected that the inflammatory phase is followed by an anti-inflammatory phase that may last several months. In patients who do not have OS, there is neither an inflammatory nor an anti-inflammatory response expected.

## 5. Discussion

If this hypothesis holds, RedoxPRF treatment has a unique position for several reasons. It has a selective effect on the overproduction of FRs and breaks its self-reinforcing cycle, which is the most upstream step in the train of events short of the causing pathology itself. It is well known that antioxidants are good for our health, but the effect of these substances on OS and IFM are not unequivocally clear. This discrepancy was named the antioxidant paradox around 2000 (for overview see [25]). Remarkably, the Nobel prize winner Szent Györgyi had already suggested a full explanation of this phenomenon four decades earlier [26]. He argues that the redox equilibrium is complex because it is not only determined by conventional mostly bivalent molecules but by sub-molecular events as well. A reaction between bivalent molecules produces two different molecules in ground state, but in the sub-molecular environment, single electrons are transferred, and this causes ongoing biological activity. Since the overproduction of FRs is clearly a sub-molecular event, a countermeasure in the same environment is a natural choice.

**Figure 3:**
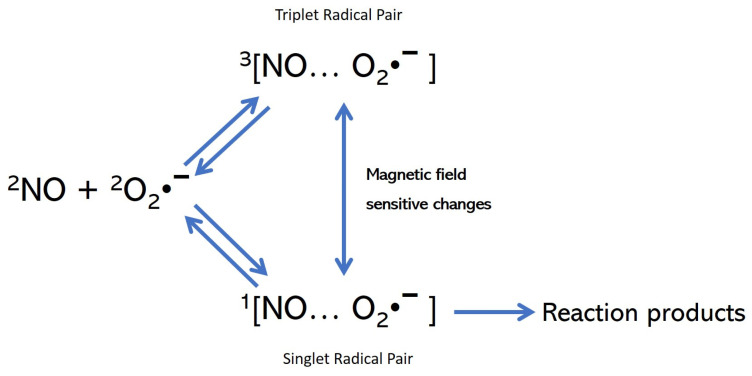
Simplified kinetic scheme showing the magnetic field-sensitive transition between the singlet and triplet state of the radical pairs (in this example, NO and O_2_•^−^). Note that the reaction can only be formed from the singlet radical pair. Figure adapted from [29] which provides also more detailed information on the effects of magnetic fields on small biologically relevant inorganic radicals.

It was earlier discussed how the electromagnetic PRF field could influence the biochemistry of redox equilibria in cells [11]. From this, it was argued that when an electromagnetic wave encounters a living organism it will induce movement in the electric charges within the body of the living organism. When there is a spatial imbalance of electric charges in a given region of space, electric dipoles are formed, as in the case of free radicals in a pathological situation. Since each electron spin has an associated magnetic moment, the interconversion and chemical fates of the singlet (S) and triplet (T) states can be influenced by internal and external magnetic fields. Therefore, an external magnetic field affects interconversion between the S and T states of the radical pair. In these conditions, an applied weak magnetic field will result in an increased transient conversion of the radical pair into the triplet state, causing triplet products to be formed more rapidly and in higher yield. Figure 3 shows a schematic example of this. Several models describing the magnetic field influence on kinetics of enzymatic reactions that involve free radical-dependent chemistry have been published (e.g., [27,28,29]). Thus, the radical pair mechanism is a plausible way in which weak magnetic field variations can affect chemical reactivity, allowing for radical pairs containing substances that can function as chemical/biological magnetic sensors. Therefore, it is possible that PRF acts on radical pair magnetic sensors affecting S–T transitions and thus exerting its therapeutic effects as a stabilizer of the redox balance and anti-inflammatory effect.

Additionally, RedoxPRF scores high in terms of safety and convenience. It is a simple procedure to perform; there are no unpleasant sensations or side effects, and with the exception of cancer patients, the need for maintenance treatment is experienced to be 2–4 months or even longer.

There are two groups of patients who might benefit. The inflammatory diseases are a large family of diseases that have OS as a common pathway in their pathology. The list contains important diseases such as stroke [30,31], diabetes type 2 [32,33], age-related macular degeneration [34,35], major depressive disorder [36,37], chronic kidney disease [38,39], COPD [40,41], osteoarthritis [42], inflammaging [43], postinfection fatigue syndrome [44], and diseases that are caused by a gene defect such as antitrypsin deficiency and epidermolysis bullosa [45]. Additionally, RedoxPRF may have an effect on the inflammatory component of grade 4 cancer. This could improve the life expectancy of some of these patients [46] and contribute to their quality of life. Finally, there might also be an effect on the immune dip that follows surgery and that may lead to complications in elderly patients [47,48].

Based on our hypothesis and observations, this looks like a new field in medicine that is worth exploring with formal studies.

The second group concerns the treatment of chronic pain. Here, RedoxPRF is a novelty because it is neither ablation nor stimulation [49]. We propose the name “functional restoration”.

## Data Availability

Not (yet) published data supporting the hypothesis in this paper can be made available to professionals upon request to the authors.
